# *N*-(3-Bromo­phen­yl)-3,4,5-trimethoxy­benzamide

**DOI:** 10.1107/S1600536809020509

**Published:** 2009-06-06

**Authors:** Aamer Saeed, Shahid Hussain, Aliya Ibrar, Michael Bolte

**Affiliations:** a Department of Chemistry, Quaid-i-Azam University, Islamabad 45320, Pakistan; b Institut für Anorganische Chemie, J. W. Goethe-Universität Frankfurt, Max-von-Laue-Str.7, 60438 Frankfurt/Main, Germany

## Abstract

In the title compound, C_16_H_16_BrNO_4_, the dihedral between the planes of the aromatic rings is 7.74 (18)°. The amide group is tilted with respect to the bromo- and meth­oxy-substituted aromatic rings by 36.3 (8) and 35.2 (8)°, respectively. The *meta*-meth­oxy groups are essentially in-plane with the aromatic ring [dihedral angles CH_3_—O—C—C = −4.6 (4) and −2.5 (4)°]. The *para*-meth­oxy group is markedly displaced from the ring plane [dihedral angle CH_3_—O—C—C = −72.5 (4)°]. The crystal packing is stabilized by N—H⋯O hydrogen bonds linking the mol­ecules into chains running along the *b* axis.

## Related literature

For related structures and general background, see: Saeed *et al.* (2009 ▶). For conformations of aromatic meth­oxy groups, see: Vande Velde *et al.* (2006 ▶).
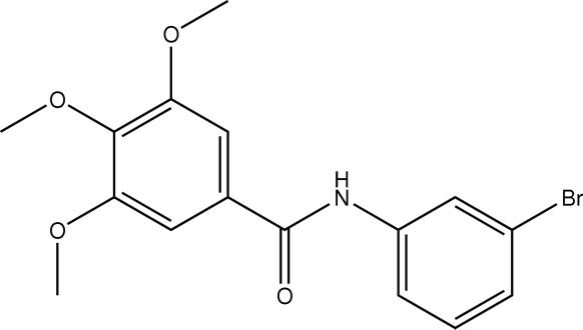


## Experimental

### 

#### Crystal data

C_16_H_16_BrNO_4_
*M* *_r_* = 366.21
Orthorhombic, 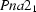

*a* = 13.3085 (8) Å
*b* = 4.9953 (3) Å
*c* = 23.4061 (12) Å
*V* = 1556.04 (15) Å^3^
*Z* = 4
Mo *K*α radiation
μ = 2.66 mm^−1^
*T* = 173 K
0.37 × 0.34 × 0.19 mm

#### Data collection

Stoe IPDS II two-circle diffractometer
Absorption correction: multi-scan [*MULABS* (Spek, 2009 ▶; Blessing, 1995 ▶)] *T*_min_ = 0.418, *T*_max_ = 0.600
11994 measured reflections
3028 independent reflections
2748 reflections with *I* > 2σ(*I*)
*R*_int_ = 0.056

#### Refinement

*R*[*F* ^2^ > 2σ(*F* ^2^)] = 0.033
*wR*(*F* ^2^) = 0.076
*S* = 0.99
3028 reflections
207 parameters
1 restraint
H atoms treated by a mixture of independent and constrained refinement
Δρ_max_ = 0.46 e Å^−3^
Δρ_min_ = −0.39 e Å^−3^
Absolute structure: Flack (1983 ▶), with 1405 Friedel pairs
Flack parameter: 0.001 (8)

### 

Data collection: *X-AREA* (Stoe & Cie, 2001 ▶); cell refinement: *X-AREA*; data reduction: *X-AREA*; program(s) used to solve structure: *SHELXS97* (Sheldrick, 2008 ▶); program(s) used to refine structure: *SHELXL97* (Sheldrick, 2008 ▶); molecular graphics: *PLATON* (Spek, 2009 ▶); software used to prepare material for publication: *SHELXL97*.

## Supplementary Material

Crystal structure: contains datablocks global, I. DOI: 10.1107/S1600536809020509/zl2213sup1.cif

Structure factors: contains datablocks I. DOI: 10.1107/S1600536809020509/zl2213Isup2.hkl

Additional supplementary materials:  crystallographic information; 3D view; checkCIF report

## Figures and Tables

**Table 1:** Hydrogen-bond geometry (Å, °)

*D*—H⋯*A*	*D*—H	H⋯*A*	*D*⋯*A*	*D*—H⋯*A*
N1—H1⋯O1^i^	0.85 (4)	2.06 (4)	2.821 (3)	149 (3)

Symmetry code: (i) 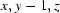.

**Fig. 1.**
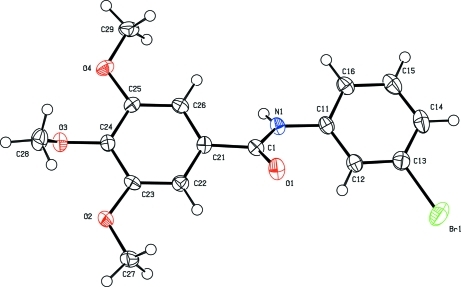
The title compound with the atom numbering scheme; displacement ellipsoids are at the 50% probability level; H atoms are drawn as small spheres of arbitrary radii.

**Fig. 2.**
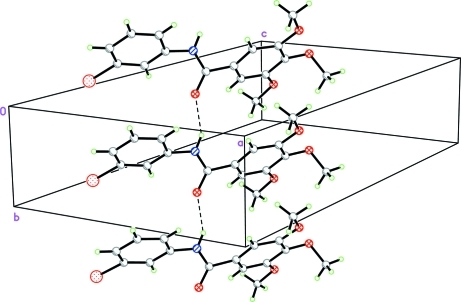
Partial packing diagram of the title compound. Hydrogen bonds are drawn as dashed lines.

## supplementary crystallographic information

### Comment

The background to this study has been described in an earlier paper on *N*-(2-chlorophenyl)-4-chlorobenzamide (Saeed *et al.*, 2009). As part of our work on the structure of benzanilides and related compounds, we report here the structure of the title compound, Fig. 1.

In the title compound, C_16_H_16_BrNO_4_, the dihedral angle between the aromatic rings is 7.74 (18)°. The amide moiety is tilted against the bromo and methoxy substituted aromatic rings by 36.3 (8)° and 35.2 (8)°, respectively. The *meta* methoxy groups are essentially in plane with the aromatic ring [dihedral angles CH_3_—O—C—C = -4.6 (4)° and -2.5 (4)°], the methoxy group in *para* position is markedly displaced from the ring plane [dihedral angle CH_3_—O—C—C = -72.5 (4)°]. This can be attributed to a combination of resonance effects, which lead for aromatic methoxy groups to being coplanar with an aromatic ring, and steric interactions, which prohibit a coplanar arrangement when more than two methoxy groups are present per benzene moiety (Vande Velde *et al.*, 2006). The crystal packing is stabilized by N—H···O hydrogen bonds linking the molecules to chains running along the *b* axis (Fig. 2).

### Experimental

3,4,5-Trimethoxybenzoyl chloride (5.4 mmol) in CHCl_3_ was treated with 3-bromoaniline (21.6 mmol) under a nitrogen atmosphere at reflux for 3 h. Upon cooling, the reaction mixture was diluted with CHCl_3_ and washed consecutively with aq 1 *M* HCl and saturated aq NaHCO_3_. The organic layer was dried over anhydrous magnesium sulfate and concentrated under reduced pressure. Crystallization of the residue in CHCl_3_ afforded the title compound (81%) as colourless needles. Anal. calcd. for C_16_H_16_BrNO_4_: C, 52.48; H, 4.40; N, 3.82%; found: C, 52.51; H, 4.36; N, 3.87

### Refinement

H atoms were located in a difference map but those bonded to C were geometrically positioned and refined using a riding model with fixed individual displacement parameters [U(H_iso_) = 1.2*U*_eq_(C) or U(H_iso_) = 1.5*U*_eq_(C_methyl_)] using a riding model with C_aromatic_—H = 0.95 Å or C_methyl_—H = 0.98 Å. The H atom bonded to N was freely refined.

### Crystal data

**Table:**

C_16_H_16_BrNO_4_	*F*(000) = 744
*M**_r_* = 366.21	*D*_x_ = 1.563 Mg m^−^^3^
Orthorhombic, *P**n**a*2_1_	Mo *K*α radiation, λ = 0.71073 Å
Hall symbol: P 2c -2n	Cell parameters from 11796 reflections
*a* = 13.3085 (8) Å	θ = 3.5–26.7°
*b* = 4.9953 (3) Å	µ = 2.66 mm^−^^1^
*c* = 23.4061 (12) Å	*T* = 173 K
*V* = 1556.04 (15) Å^3^	Plate, colourless
*Z* = 4	0.37 × 0.34 × 0.19 mm

### Data collection

**Table:**

Stoe IPDS II two-circle diffractometer	3028 independent reflections
Radiation source: fine-focus sealed tube	2748 reflections with *I* > 2σ(*I*)
graphite	*R*_int_ = 0.056
ω scans	θ_max_ = 26.3°, θ_min_ = 3.5°
Absorption correction: multi-scan [*MULABS* (Spek, 2009; Blessing, 1995)]	*h* = −16→16
*T*_min_ = 0.418, *T*_max_ = 0.600	*k* = −6→6
11994 measured reflections	*l* = −29→26

### Refinement

**Table:**

Refinement on *F*^2^	Hydrogen site location: inferred from neighbouring sites
Least-squares matrix: full	H atoms treated by a mixture of independent and constrained refinement
*R*[*F*^2^ > 2σ(*F*^2^)] = 0.033	*w* = 1/[σ^2^(*F*_o_^2^) + (0.0493*P*)^2^] where *P* = (*F*_o_^2^ + 2*F*_c_^2^)/3
*wR*(*F*^2^) = 0.076	(Δ/σ)_max_ < 0.001
*S* = 0.99	Δρ_max_ = 0.46 e Å^−^^3^
3028 reflections	Δρ_min_ = −0.39 e Å^−^^3^
207 parameters	Extinction correction: *SHELXL97* (Sheldrick, 2008), Fc^*^=kFc[1+0.001xFc^2^λ^3^/sin(2θ)]^-1/4^
1 restraint	Extinction coefficient: 0.0168 (10)
Primary atom site location: structure-invariant direct methods	Absolute structure: Flack (1983), with 1405 Friedel pairs
Secondary atom site location: difference Fourier map	Flack parameter: 0.001 (8)

### Special details

**Table:**

Geometry. All e.s.d.'s (except the e.s.d. in the dihedral angle between two l.s. planes) are estimated using the full covariance matrix. The cell e.s.d.'s are taken into account individually in the estimation of e.s.d.'s in distances, angles and torsion angles; correlations between e.s.d.'s in cell parameters are only used when they are defined by crystal symmetry. An approximate (isotropic) treatment of cell e.s.d.'s is used for estimating e.s.d.'s involving l.s. planes.
Refinement. Refinement of *F*^2^ against ALL reflections. The weighted *R*-factor *wR* and goodness of fit *S* are based on *F*^2^, conventional *R*-factors *R* are based on *F*, with *F* set to zero for negative *F*^2^. The threshold expression of *F*^2^ > σ(*F*^2^) is used only for calculating *R*-factors(gt) *etc*. and is not relevant to the choice of reflections for refinement. *R*-factors based on *F*^2^ are statistically about twice as large as those based on *F*, and *R*- factors based on ALL data will be even larger.

### Fractional atomic coordinates and isotropic or equivalent isotropic displacement parameters (Å^2^)

**Table:**

	*x*	*y*	*z*	*U*_iso_*/*U*_eq_	
Br1	0.19897 (2)	0.79946 (7)	0.12371 (2)	0.04356 (13)	
N1	0.52764 (18)	0.5080 (5)	0.23414 (11)	0.0203 (5)	
H1	0.546 (3)	0.351 (8)	0.2441 (17)	0.023 (9)*	
O1	0.51118 (18)	0.9515 (4)	0.25246 (10)	0.0285 (5)	
O2	0.62891 (16)	0.9862 (4)	0.46087 (9)	0.0276 (4)	
O3	0.78413 (15)	0.6364 (4)	0.46308 (10)	0.0259 (4)	
O4	0.82971 (16)	0.3369 (4)	0.37342 (10)	0.0276 (5)	
C1	0.5457 (2)	0.7303 (5)	0.26493 (13)	0.0204 (6)	
C11	0.4666 (2)	0.4924 (5)	0.18432 (13)	0.0220 (6)	
C12	0.3799 (2)	0.6447 (6)	0.17818 (13)	0.0246 (6)	
H12	0.3613	0.7724	0.2063	0.030*	
C13	0.3216 (2)	0.6057 (6)	0.13014 (16)	0.0279 (6)	
C14	0.3467 (3)	0.4244 (7)	0.08727 (14)	0.0334 (7)	
H14	0.3060	0.4043	0.0542	0.040*	
C15	0.4334 (3)	0.2740 (7)	0.09471 (15)	0.0332 (7)	
H15	0.4520	0.1466	0.0665	0.040*	
C16	0.4931 (3)	0.3066 (6)	0.14241 (13)	0.0270 (6)	
H16	0.5523	0.2022	0.1467	0.032*	
C21	0.6099 (2)	0.6961 (5)	0.31655 (12)	0.0201 (5)	
C22	0.5883 (2)	0.8619 (5)	0.36327 (13)	0.0220 (6)	
H22	0.5343	0.9859	0.3614	0.026*	
C23	0.6460 (2)	0.8435 (5)	0.41199 (12)	0.0206 (6)	
C24	0.7280 (2)	0.6655 (5)	0.41433 (13)	0.0210 (6)	
C25	0.7487 (2)	0.5016 (5)	0.36721 (13)	0.0201 (5)	
C26	0.6890 (2)	0.5141 (5)	0.31853 (13)	0.0213 (6)	
H26	0.7021	0.3997	0.2870	0.026*	
C27	0.5500 (2)	1.1804 (6)	0.45934 (14)	0.0279 (6)	
H27A	0.5610	1.3033	0.4273	0.042*	
H27B	0.5495	1.2817	0.4952	0.042*	
H27C	0.4853	1.0895	0.4545	0.042*	
C28	0.8483 (3)	0.8582 (7)	0.47549 (15)	0.0337 (7)	
H28A	0.9001	0.8727	0.4459	0.051*	
H28B	0.8805	0.8308	0.5127	0.051*	
H28C	0.8086	1.0232	0.4764	0.051*	
C29	0.8502 (2)	0.1607 (6)	0.32691 (15)	0.0287 (7)	
H29A	0.7910	0.0496	0.3194	0.043*	
H29B	0.9074	0.0456	0.3366	0.043*	
H29C	0.8664	0.2659	0.2928	0.043*	

### Atomic displacement parameters (Å^2^)

**Table:**

	*U*^11^	*U*^22^	*U*^33^	*U*^12^	*U*^13^	*U*^23^
Br1	0.02770 (15)	0.0536 (2)	0.0494 (2)	0.00300 (13)	−0.0092 (2)	0.0107 (3)
N1	0.0287 (12)	0.0138 (10)	0.0184 (12)	−0.0002 (9)	−0.0028 (10)	−0.0013 (9)
O1	0.0429 (12)	0.0152 (9)	0.0273 (11)	0.0026 (8)	−0.0104 (10)	−0.0011 (8)
O2	0.0303 (11)	0.0332 (10)	0.0192 (10)	0.0084 (8)	−0.0025 (9)	−0.0069 (9)
O3	0.0274 (10)	0.0289 (10)	0.0214 (11)	−0.0008 (8)	−0.0053 (8)	0.0023 (8)
O4	0.0232 (10)	0.0286 (11)	0.0311 (12)	0.0097 (8)	−0.0021 (9)	−0.0039 (9)
C1	0.0237 (13)	0.0175 (12)	0.0199 (14)	−0.0026 (9)	0.0012 (11)	0.0009 (10)
C11	0.0280 (14)	0.0206 (11)	0.0176 (14)	−0.0031 (11)	0.0002 (11)	0.0026 (11)
C12	0.0277 (15)	0.0248 (13)	0.0212 (14)	−0.0013 (11)	−0.0001 (12)	0.0019 (11)
C13	0.0250 (12)	0.0337 (13)	0.0251 (18)	−0.0052 (10)	0.0008 (13)	0.0053 (14)
C14	0.0352 (17)	0.0428 (17)	0.0223 (16)	−0.0146 (14)	−0.0052 (13)	0.0034 (14)
C15	0.0402 (19)	0.0357 (17)	0.0235 (17)	−0.0072 (14)	0.0011 (14)	−0.0058 (13)
C16	0.0326 (16)	0.0253 (14)	0.0232 (16)	−0.0008 (12)	0.0003 (12)	−0.0039 (11)
C21	0.0225 (14)	0.0169 (11)	0.0208 (14)	−0.0018 (10)	−0.0025 (10)	0.0014 (11)
C22	0.0244 (14)	0.0184 (12)	0.0232 (15)	0.0018 (10)	0.0007 (12)	−0.0005 (10)
C23	0.0242 (14)	0.0197 (13)	0.0178 (14)	−0.0015 (10)	0.0040 (11)	−0.0035 (11)
C24	0.0215 (13)	0.0229 (13)	0.0186 (14)	−0.0018 (10)	−0.0016 (11)	0.0009 (11)
C25	0.0181 (12)	0.0185 (13)	0.0238 (15)	−0.0001 (10)	0.0005 (11)	0.0021 (10)
C26	0.0243 (14)	0.0191 (12)	0.0205 (14)	−0.0010 (10)	0.0029 (11)	−0.0019 (10)
C27	0.0324 (16)	0.0244 (14)	0.0269 (16)	0.0064 (12)	0.0015 (13)	−0.0055 (12)
C28	0.0318 (17)	0.0352 (17)	0.0341 (19)	−0.0032 (14)	−0.0116 (14)	−0.0036 (14)
C29	0.0296 (16)	0.0257 (15)	0.0308 (17)	0.0070 (12)	0.0062 (13)	−0.0021 (13)

### Geometric parameters (Å, °)

**Table:**

Br1—C13	1.903 (3)	C15—H15	0.9500
N1—C1	1.346 (4)	C16—H16	0.9500
N1—C11	1.423 (4)	C21—C26	1.392 (4)
N1—H1	0.85 (4)	C21—C22	1.401 (4)
O1—C1	1.232 (3)	C22—C23	1.378 (4)
O2—C23	1.367 (3)	C22—H22	0.9500
O2—C27	1.431 (3)	C23—C24	1.409 (4)
O3—C24	1.371 (4)	C24—C25	1.401 (4)
O3—C28	1.429 (4)	C25—C26	1.391 (4)
O4—C25	1.363 (3)	C26—H26	0.9500
O4—C29	1.426 (4)	C27—H27A	0.9800
C1—C21	1.490 (4)	C27—H27B	0.9800
C11—C12	1.389 (4)	C27—H27C	0.9800
C11—C16	1.396 (4)	C28—H28A	0.9800
C12—C13	1.380 (5)	C28—H28B	0.9800
C12—H12	0.9500	C28—H28C	0.9800
C13—C14	1.393 (5)	C29—H29A	0.9800
C14—C15	1.388 (5)	C29—H29B	0.9800
C14—H14	0.9500	C29—H29C	0.9800
C15—C16	1.379 (4)		
			
C1—N1—C11	125.9 (2)	C21—C22—H22	120.3
C1—N1—H1	124 (2)	O2—C23—C22	124.4 (3)
C11—N1—H1	110 (2)	O2—C23—C24	115.2 (2)
C23—O2—C27	117.1 (2)	C22—C23—C24	120.4 (3)
C24—O3—C28	114.4 (2)	O3—C24—C25	119.1 (3)
C25—O4—C29	116.3 (2)	O3—C24—C23	121.4 (2)
O1—C1—N1	123.2 (3)	C25—C24—C23	119.4 (3)
O1—C1—C21	120.6 (2)	O4—C25—C26	124.5 (3)
N1—C1—C21	116.2 (2)	O4—C25—C24	115.1 (3)
C12—C11—C16	120.1 (3)	C26—C25—C24	120.4 (2)
C12—C11—N1	121.9 (3)	C25—C26—C21	119.3 (3)
C16—C11—N1	117.9 (3)	C25—C26—H26	120.4
C13—C12—C11	118.3 (3)	C21—C26—H26	120.4
C13—C12—H12	120.8	O2—C27—H27A	109.5
C11—C12—H12	120.8	O2—C27—H27B	109.5
C12—C13—C14	122.9 (3)	H27A—C27—H27B	109.5
C12—C13—Br1	118.4 (3)	O2—C27—H27C	109.5
C14—C13—Br1	118.7 (2)	H27A—C27—H27C	109.5
C15—C14—C13	117.5 (3)	H27B—C27—H27C	109.5
C15—C14—H14	121.3	O3—C28—H28A	109.5
C13—C14—H14	121.3	O3—C28—H28B	109.5
C16—C15—C14	121.1 (3)	H28A—C28—H28B	109.5
C16—C15—H15	119.5	O3—C28—H28C	109.5
C14—C15—H15	119.5	H28A—C28—H28C	109.5
C15—C16—C11	120.1 (3)	H28B—C28—H28C	109.5
C15—C16—H16	119.9	O4—C29—H29A	109.5
C11—C16—H16	119.9	O4—C29—H29B	109.5
C26—C21—C22	121.0 (3)	H29A—C29—H29B	109.5
C26—C21—C1	122.4 (2)	O4—C29—H29C	109.5
C22—C21—C1	116.6 (2)	H29A—C29—H29C	109.5
C23—C22—C21	119.5 (3)	H29B—C29—H29C	109.5
C23—C22—H22	120.3		
			
C11—N1—C1—O1	−0.9 (5)	C27—O2—C23—C22	−4.6 (4)
C11—N1—C1—C21	178.3 (3)	C27—O2—C23—C24	176.5 (2)
C1—N1—C11—C12	−35.8 (4)	C21—C22—C23—O2	−176.9 (3)
C1—N1—C11—C16	147.8 (3)	C21—C22—C23—C24	1.9 (4)
C16—C11—C12—C13	0.3 (4)	C28—O3—C24—C25	111.7 (3)
N1—C11—C12—C13	−176.0 (3)	C28—O3—C24—C23	−72.5 (4)
C11—C12—C13—C14	−1.2 (4)	O2—C23—C24—O3	1.4 (4)
C11—C12—C13—Br1	176.5 (2)	C22—C23—C24—O3	−177.5 (3)
C12—C13—C14—C15	1.5 (5)	O2—C23—C24—C25	177.1 (2)
Br1—C13—C14—C15	−176.2 (2)	C22—C23—C24—C25	−1.8 (4)
C13—C14—C15—C16	−1.0 (5)	C29—O4—C25—C26	−2.5 (4)
C14—C15—C16—C11	0.2 (5)	C29—O4—C25—C24	177.7 (3)
C12—C11—C16—C15	0.2 (4)	O3—C24—C25—O4	−4.4 (4)
N1—C11—C16—C15	176.6 (3)	C23—C24—C25—O4	179.8 (2)
O1—C1—C21—C26	−146.1 (3)	O3—C24—C25—C26	175.8 (3)
N1—C1—C21—C26	34.6 (4)	C23—C24—C25—C26	0.0 (4)
O1—C1—C21—C22	32.5 (4)	O4—C25—C26—C21	−178.2 (2)
N1—C1—C21—C22	−146.8 (3)	C24—C25—C26—C21	1.6 (4)
C26—C21—C22—C23	−0.3 (4)	C22—C21—C26—C25	−1.5 (4)
C1—C21—C22—C23	−178.9 (2)	C1—C21—C26—C25	177.1 (3)

### Hydrogen-bond geometry (Å, °)

**Table:**

*D*—H···*A*	*D*—H	H···*A*	*D*···*A*	*D*—H···*A*
N1—H1···O1^i^	0.85 (4)	2.06 (4)	2.821 (3)	149 (3)

Symmetry codes: (i) *x*, *y*−1, *z*.
